# Radiotherapy plus immunotherapy with or without chemotherapy improves survival in elderly esophageal squamous cell carcinoma: a real-world comorbidity-stratified study

**DOI:** 10.3389/fonc.2026.1748355

**Published:** 2026-05-04

**Authors:** Hong jian Ren, Qi lin Ren, Zhi juan Zhang, Jun jie Lei, Ning Xu

**Affiliations:** 1 Fenyang Hospital of Shanxi Province, Fenyang, China; 2 School of Stomatology, Tongji University, Shanghai, China

**Keywords:** CIRS-G, comorbidity stratification, elderly patients, esophageal squamous cell carcinoma, immunotherapy, radiotherapy, real-world study

## Abstract

**Objective:**

This real-world study explores the efficacy and safety of radiotherapy combined with immunotherapy-based regimens in elderly patients (≥70 years) with esophageal squamous cell carcinoma (ESCC), stratified by the Cumulative Illness Rating Scale--Geriatric (CIRS-G). Given the exploratory nature and modest sample size, findings should be interpreted as hypothesis-generating.

**Methods:**

We conducted a single-center retrospective analysis of 87 elderly ESCC patients receiving intensity-modulated radiotherapy, categorized into three groups: radiotherapy alone (RT, n=49), concurrent chemoradiotherapy (CRT, n=18), and radiotherapy combined with immunotherapy ± chemotherapy (RIT ± CT, n=20). The primary endpoint was overall survival (OS). Propensity score matching (PSM) and multivariable Cox regression were performed.

**Results:**

With a median follow-up of 37 months, multivariable Cox regression analysis suggested that the RIT ± CT regimen was associated with a reduced mortality risk versus RT (HR = 0.341, 95% CI: 0.130–0.896, P = 0.029). PSM confirmed this survival advantage (OS: HR = 0.42, P = 0.028). Patients with a medium CIRS-G burden (scores 4–6) demonstrated the most significant OS benefit from this multimodal strategy. Grade ≥3 TRAEs were highest in the RIT ± CT group (55.0%), with no treatment-related deaths.

**Conclusion:**

The integration of immunotherapy-based regimens into radiotherapy shows an association with improved survival in elderly ESCC patients with a manageable safety profile. The CIRS-G score may help identify optimal candidates. These exploratory findings require validation in larger, prospective multicenter studies. The independent contribution of immunotherapy alone cannot be determined from this study.

## 1 Introduction

Esophageal carcinoma remains one of the most aggressive malignancies globally, with incidence strongly age-related and median diagnosis age of 68 years (1, 2). The growing aging population presents unique challenges in managing elderly esophageal carcinoma patients, who often exhibit physiological decline, multiple comorbidities, and immunosenescence, limiting tolerance for traditional intensive therapies (3, 4).

Immune checkpoint inhibitors have transformed the ESCC treatment landscape. Phase III trials including KEYNOTE-590, CheckMate 648, CheckMate 649, and ESCORT-1st established PD-1 inhibitors plus chemotherapy efficacy in advanced esophageal cancer (5–8). In the second-line setting, the ATTRACTION-3 trial demonstrated that nivolumab monotherapy significantly improved overall survival compared with chemotherapy (9). Radiotherapy induces immunogenic cell death, releasing tumor antigens, whereas ICIs reverse T-cell suppression. This synergy creates a potential “*in situ* vaccine” effect (8, 10). However, patients ≥70 years were substantially underrepresented (<15%) in these pivotal trials, necessitating real-world validation in this population.

Given elderly patient heterogeneity, chronological age alone inadequately guides treatment. The CIRS-G, a validated tool that systematically assesses severity across 14 organ systems, provides more comprehensive risk and prognosis evaluation (11, 12). This study innovatively integrates CIRS-G stratification into the elderly ESCC treatment framework, evaluating the efficacy and safety of multimodal treatment strategies involving radiotherapy combined with immunotherapy-based regimens across comorbidity burdens. Importantly, most patients in the RIT ± CT group also received chemotherapy, so the observed benefits reflect a multimodal strategy rather than immunotherapy alone.

## 2 Materials and methods

### 2.1 Study design and patient population

This single-center retrospective observational cohort study complied with the Declaration of Helsinki and was approved by the Ethics Committee of Shanxi Fenyang Hospital (Approval No.: 2023054). Informed consent was waived due to the retrospective design.

We consecutively screened ESCC patients receiving IMRT between October 2017 and October 2024. Inclusion criteria: (1) age ≥70 years; (2) pathologically confirmed ESCC; (3) ECOG performance status 0–1. Exclusion criteria: (1) prior radical esophagectomy or thoracic radiotherapy; (2) active autoimmune diseases/immunosuppressive therapy; (3) distant metastases other than supraclavicular lymph node metastasis; (4) critically incomplete clinical data.

Finally, 87 patients were included and categorized:

- Radiotherapy alone (RT) group (n=49): IMRT only.
- Chemoradiotherapy (CRT) group (n=18): IMRT with concurrent or sequential platinum-based doublet chemotherapy.
- Radiotherapy combined with immunotherapy ± chemotherapy (RIT ± CT) group (n=20): IMRT combined with PD-1 inhibitors (camrelizumab, tislelizumab, or toripalimab), with 17 patients (85%) also receiving chemotherapy.

Given the exploratory nature of this real-world study and the modest sample size, particularly in the RIT ± CT group (n=20), all inferential statistics are intended to generate hypotheses rather than provide definitive conclusions. Results should be interpreted with caution and require external validation.

### 2.2 Treatment protocols

All treatment decisions were made by a multidisciplinary team.

Radiotherapy: All patients received IMRT using helical CT simulation with supine immobilization. Target volumes were delineated using thoracic contrast-enhanced CT, upper GI radiography, endoscopy/ultrasound, and/or PET-CT. GTV included primary tumor (GTVp) and involved nodes (GTVnd). CTV expanded GTVp 0.6 cm radially (respecting anatomical barriers) and 2–3 cm craniocaudally; GTVnd expanded 0.5 cm radially. PTV added a 0.5-cm margin to CTV. Involved-field or elective nodal irradiation was used. The prescribed dose was 50–69.96 Gy in 1.8–2.12-Gy fractions. Treatment was delivered via an Elekta linear accelerator, ensuring 95% PTV coverage by the 95% isodose line. Organ-at-risk constraints followed QUANTEC guidelines (13).

Systemic therapy and treatment sequencing: Chemotherapy comprised taxane- or fluorouracil-based doublets with platinum, with doses adjusted for renal function, hematologic parameters, and overall condition. Immunotherapy involved PD-1 inhibitors (camrelizumab, tislelizumab, or toripalimab).

The choice of PD-1 inhibitor and the decision to add chemotherapy were made by the multidisciplinary team based on tumor stage, performance status, organ function, and expected tolerance. Chemotherapy was added (17/20 patients) when clinically feasible and deemed necessary for rapid tumor debulking. In the RIT ± CT group (n=20), 17 patients received induction immunotherapy plus chemotherapy followed by sequential radiotherapy and subsequent immunotherapy consolidation; 1 patient received concurrent radiotherapy plus immunotherapy; and 2 patients received sequential immunotherapy after radiotherapy. In the CRT group (n=18), chemotherapy was administered concurrently with radiotherapy in 3 patients and sequentially in 15 patients.

### 2.3 Comorbidity assessment and stratification

Two independent geriatricians assessed comorbidities using CIRS-G, evaluating 14 organ systems scored 0 (no impairment) to 4 (extremely severe/life-threatening). Total score defined overall comorbidity burden. Interrater reliability was excellent (Cohen’s κ=0.81, P<0.001). A senior physician arbitrated discrepancies involving ≥2 organ systems or total score difference ≥5 points. Patients were stratified into low (0–3), medium (4–6), and high (≥7) comorbidity burden groups per established criteria (11).

### 2.4 Study endpoints and evaluation criteria

The primary endpoint was overall survival (OS), defined as the time from treatment initiation (first dose of radiotherapy or systemic therapy) to death from any cause, with survivors censored at the last follow-up date. Secondary endpoints included progression-free survival (PFS), defined as the time from treatment initiation to disease progression (assessed by RECIST 1.1) or death from any cause, and patterns of failure and treatment safety. Treatment-related adverse events (TRAEs) were graded according to the RTOG acute radiation morbidity criteria.

### 2.5 Follow-up management

Patients were followed every 3 months via structured telephone interviews and outpatient reviews until death or study cutoff (October 2024). Loss to follow-up was censored in survival analyses.

### 2.6 Statistical analysis

SPSS 27.0 and R 4.0 were used. Baseline characteristics were compared using t-tests, chi-square tests, or Fisher’s exact tests. Survival analysis was conducted using the Kaplan–Meier method with the log-rank test. Prognostic factors were analyzed using univariate and multivariate Cox proportional hazard models.

Propensity score matching: Because the CRT group had a small sample size and was not the focus of the primary comparison, we performed 1:1 nearest-neighbor matching (caliper=0.2) only between the RT and RIT ± CT groups, including age, gender, BMI, tumor length, location, clinical stage, T stage, nodal status, and CIRS-G score. This yielded 18 pairs (36 patients). The CRT group was retained in descriptive analyses but not in the matched survival comparison. All P-values were two-sided; P<0.05 was considered statistically significant for exploratory hypothesis generation. Due to the limited number of events, particularly in the RIT ± CT group, multivariable Cox regression results should be interpreted as provisional.

## 3 Results

### 3.1 Patient characteristics

The cohort’s median age was 76 years (range: 70–91), comprising 52 men (59.8%) and 35 women (40.2%). Most patients (96.6%, 84/87) had ≥1 comorbidity. The most prevalent were hypertension (72.4%), cardiovascular disease (58.6%), chronic respiratory disease (44.8%), and diabetes (21.8%). Baseline characteristics were comparable across the three treatment groups, except for age distribution, which differed significantly (P = 0.004, **Table 1**). The median CIRS-G score was 6 (range 1–11). CIRS-G stratification distributed 24 (27.6%), 36 (41.4%), and 27 (31.0%) patients into low-, medium-, and high-burden groups, respectively, with no significant distribution difference across treatment groups. According to the AJCC 8th edition, the stage IV patients included in this cohort all had isolated supraclavicular lymph node metastasis without other distant organ involvement. The CIRS-G score distribution and stratification are presented in **Tables 2**, **3**, and baseline clinical characteristics in **Table 1**.

**Table 1:** Clinical characteristics of 87 elderly patients with ESCC.

Grouping	Variable	Total (n=87)	RT (n=49)	CRT (n=18)	RIT ± CT (n=20)	χ²/Fisher value	P-value
Age	70–74	30	11	12	7	15.534	0.004
	75–79	28	15	4	9		
	80+	29	23	2	4		
Gender	Male	52	31	9	12	0.964	0.618
	Female	35	18	9	8		
BMI	Normal weight	53	30	9	14		0.399*
	Overweight	20	9	6	5		
	Underweight	14	10	3	1		
Length	≤5 cm	44	27	8	9	0.921	0.631
	>5 cm	43	22	10	11		
Tumor location	Lower thoracic	25	12	5	8		0.737*
	Middle thoracic	43	25	10	8		
	Upper thoracic	19	12	3	4		
Stage simplified	Early (I-II)	39	21	10	8	1.103	0.576
	Advanced (III-IV)	48	28	8	12		
CIRS_G score	0–3	24	16	4	4		0.616*
	4–6	36	21	7	8		
	7–11	27	12	7	8		
T period	T1+T2	29	14	8	7	1.525	0.466
	T3+T4	58	35	10	13		
Lymph node metastasis	No	36	22	9	5	3.014	0.222
	Yes	51	27	9	15		

*Fisher’s exact test was used when expected cell counts were <5.

**Table 2:** CIRS-G score distribution in 87 elderly patients with ESCC.

Item	RT group (n=49)	CRT group (n=18)	RIT ± CT group (n=20)	Total (n=87)
CIRS-G≥2	45	15	19	79
CIRS-G≥3	35	14	17	66
CIRS-G≥4	31	14	16	61
CIRS-G≥5	21	10	12	43
CIRS-G≥6	18	8	11	37
CIRS-G≥7	12	7	8	27
CIRS-G≥8	10	6	4	20
CIRS-G≥9	6	6	2	14
CIRS-G≥10	6	1	1	8
CIRS-G≥11	3	0	0	3

**Table 3:** CIRS-G stratification of 87 elderly patients with ESCC.

Item	RT group (n=49)	CRT group (n=18)	RIT ± CT group (n=20)	Total (n=87)	χ² value	P-value
Low burden (score 0–3)	16 (32.7%)	4 (22.2%)	4 (20.0%)	24	1.466	0.48
Medium burden (score 4–6)	21 (42.9%)	7 (38.9%)	8 (40.0%)	36	0.106	0.95
High burden (score ≥7)	12 (24.5%)	7 (38.9%)	8 (40.0%)	27	2.25	0.325

### 3.2 Treatment completion and parameters

All patients received radiotherapy, and 72 (82.8%) completed the prescribed course. Median radiation dose was 60 Gy (range 6–69.96), and 71 (81.6%) received 50–60 Gy. There were 15 (17.2%) who did not complete radiotherapy due to toxicity intolerance, disease progression, or patient preference. Most patients (81.6%, 71/87) received involved-field irradiation. Radiation dose and target volume distribution did not differ significantly among groups. Detailed radiotherapy parameters are summarized in **Table 4**.

**Table 4:** Radiotherapy characteristics of 87 elderly patients with ESCC.

Grouping	Item	RT (n=49)	CRT (n=18)	RIT ± CT (n=20)	Total (n=87)	χ²/Fisher value	P-value
Radiation target volume	ENI	10 (20.4%)	3 (16.7%)	3 (15%)	16 (18.4%)	0.454	0.797
	IFI	39 (79.6%)	15 (83.3%)	17 (85%)	71 (81.6%)		
Radiation dose	≤50 Gy	11 (22.4%)	1 (5.6%)	3 (15%)	15 (17.2%)	3.209	0.201
	>50 Gy	38 (77.5%)	17 (94.4%)	17 (85%)	72 (82.7%)		

ENI, elective nodal irradiation; IFI, involved-field irradiation.

### 3.3 Survival outcomes

Definition note: Both OS and PFS were calculated from treatment start. Loss to follow-up was censored.

**Figure 1:**
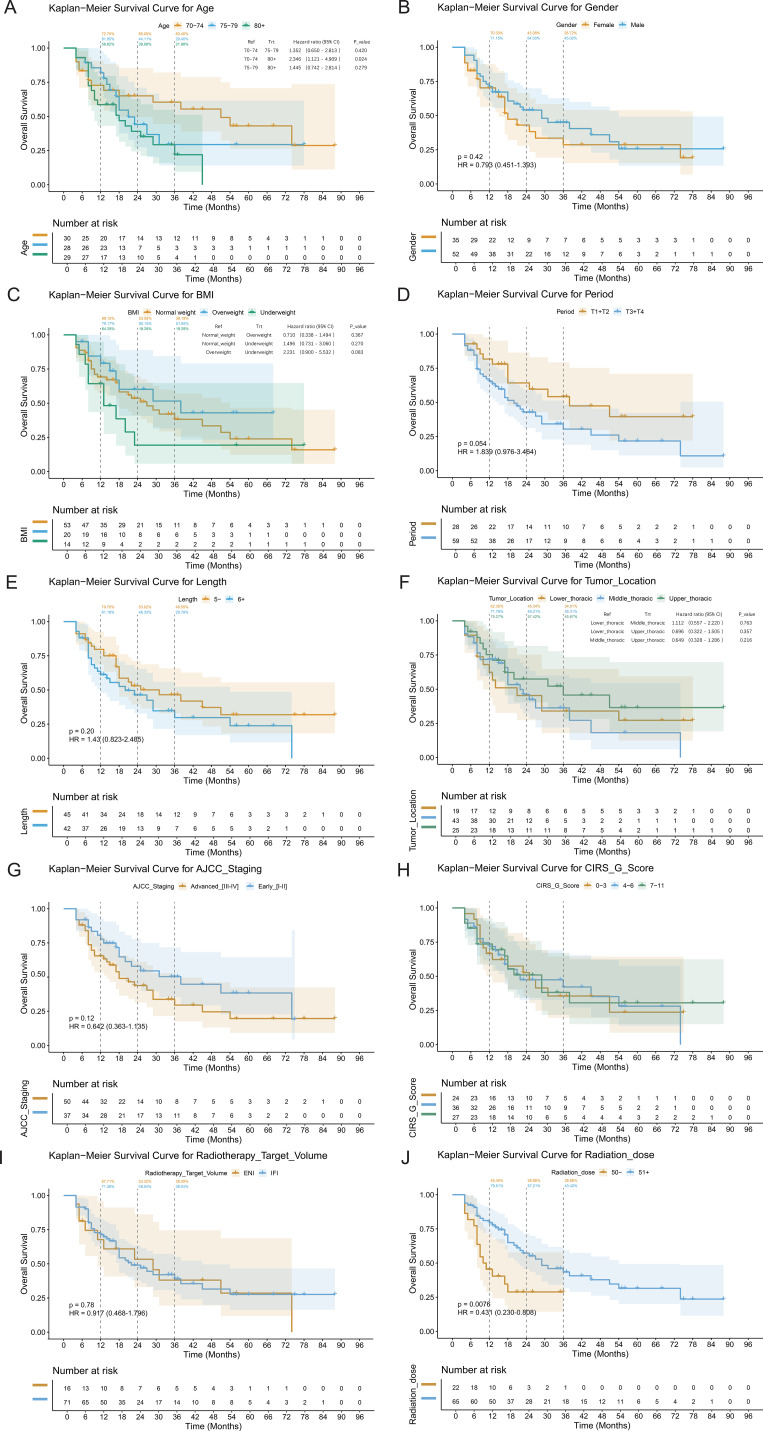
Overall survival curves of elderly ESCC patients stratified by various clinicopathological variables. Kaplan–Meier survival curves stratified by clinicopathological variables. **(A)** Age, **(B)** gender, **(C)** BMI, **(D)** T stage, **(E)** tumor length, **(F)** tumor location, **(G)** clinical stage, **(H)** CIRS-G burden, **(I)** radiotherapy target volume (IFI vs. ENI), and **(J)** radiation dose. Log-rank P-values are shown.

**Figure 2:**
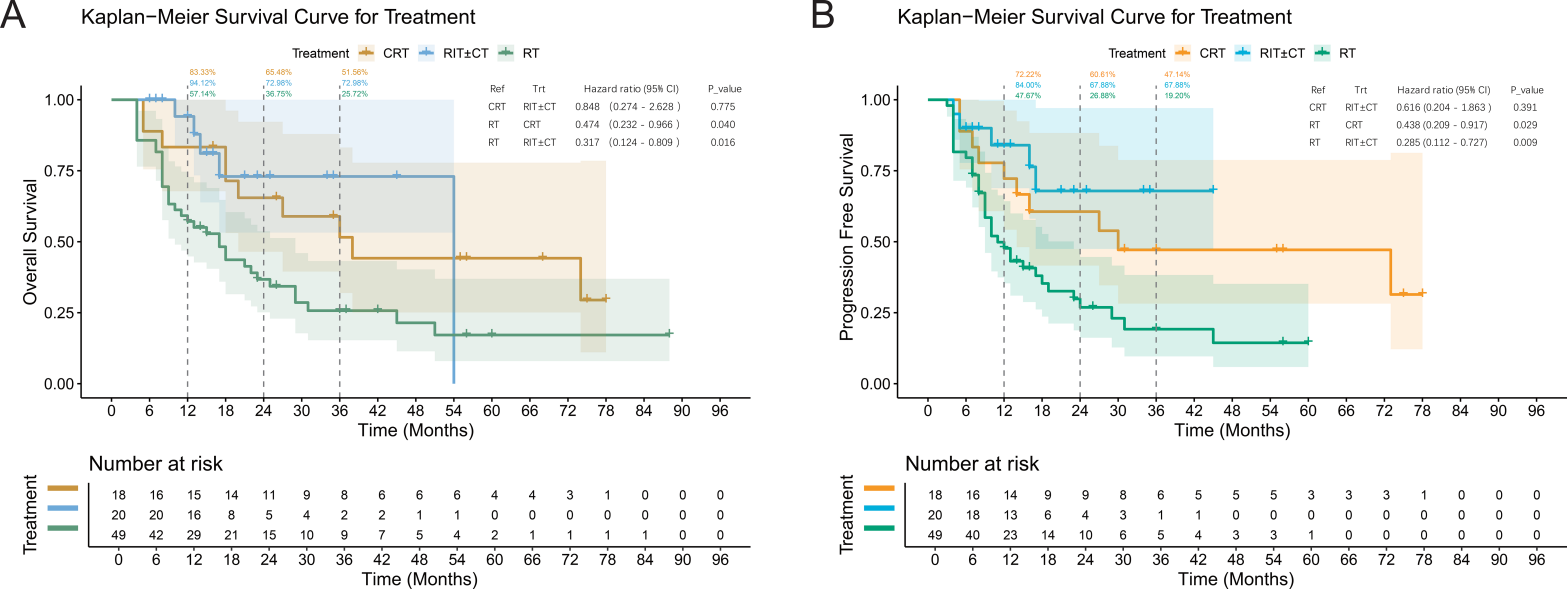
Overall survival **(A)** and progression-free survival **(B)** curves of elderly ESCC patients stratified by treatment group. Survival analysis by treatment group. **(A)** Overall survival (OS) and **(B)** progression-free survival (PFS) curves for the RT in green, CRT in yellow, RIT±CT in blue groups. Log-rank P = 0.009 (OS) and P = 0.004 (PFS).

The median follow-up was 37 months (range: 4–84). Median OS was 38.5 months, and median PFS was 19.2 months. The RIT ± CT group (predominantly receiving triplet therapy) showed the most favorable OS, with 1-, 2-, and 3-year rates of 94.12%, 72.98%, and 72.98%, respectively, compared with the CRT (83.33%, 65.48%, 51.56%) and RT groups (57.14%, 36.75%, 25.72%). PFS differed significantly among groups (log-rank P = 0.004). Multivariable Cox regression suggested that RIT ± CT was an independent predictor of improved OS versus RT (adjusted HR = 0.341, 95% CI: 0.130–0.896, P = 0.029). Given the small sample size (n=20 in the RIT ± CT group) and the limited number of events, this finding is exploratory and requires confirmation in larger cohorts. The wide confidence interval underscores the imprecision of the estimate. CRT showed a non-significant survival trend (adjusted HR = 0.537, 95% CI: 0.247–1.168, P = 0.117). Higher radiation dose was an independent protective factor (adjusted HR = 0.962 per 1 Gy increase, 95% CI: 0.940–0.984, P<0.001), whereas tumor length >5 cm was an independent mortality risk factor (adjusted HR = 1.294, 95% CI: 1.081–1.549, P = 0.005). Age, gender, AJCC stage, tumor location, and total CIRS-G score lacked independent prognostic significance. The corresponding survival curves and detailed analysis are presented in **Figures 1**, **2**, **Table 5**.

**Table 5:** Analysis of prognostic factors.

Characteristics	Total (N)	Univariate analysis HR (95% CI)	P value	Multivariate analysis HR (95% CI)	P value
Age	87	1.062 (1.010–1.116)	0.018	1.050 (0.988–1.115)	0.115
Male	52	Reference			
Female	35	0.793 (0.451–1.393)	0.420		
BMI	87	0.925 (0.839–1.020)	0.118	0.943 (0.857–1.038)	0.230
AJCC stages I-II	37	Reference		Reference	
AJCC stages III-IV	50	1.839 (0.976–3.464)	0.054	1.097 (0.562–2.144)	0.786
Tumor length	87	1.43 (0.823–2.485)	0.20	1.294 (1.081–1.549)	0.005
Tumor location lower thoracic	19	Reference			
Tumor location middle thoracic	43	1.112 (0.557–2.220)	0.763		
Tumor location upper thoracic	25	0.696 (0.322–1.505)	0.357		
Treatment RT	49	Reference		Reference	
Treatment CRT	18	0.474 (0.232–0.966)	0.040	0.537 (0.247–1.168)	0.117
Treatment RIT ± CT	20	0.317 (0.124–0.809)	0.016	0.341 (0.130–0.896)	0.029
CIRS-G score	87	1.031 (0.940–1.132)	0.514		
Radiotherapy target volume IFI	71	Reference			
Radiotherapy target volume ENI	16	0.917 (0.468–1.796)	0.78		
Radiation dose*	87	0.431 (0.230–0.808)†	0.0076	0.962 (0.940–0.984)‡	<0.001

*Radiation dose: categorical (>50 Gy vs. ≤50 Gy) in univariate analysis; continuous (per 1 Gy) in multivariate analysis. ^†^HR for >50 Gy vs. ≤50 Gy. ^‡^HR per 1 Gy increase.

### 3.4 Patterns of failure

We analyzed the first site of disease progression to assess disease control across treatment groups. The overall failure rate was highest in the RT group (57.1%, 28/49), followed by the CRT (44.4%, 8/18) and RIT ± CT groups (35.0%, 7/20). The locoregional recurrence rates were 38.8% (19/49) in the RT group, 27.8% (5/18) in the CRT group, and 25.0% (5/20) in the RIT ± CT group. The distant metastasis rates were 30.6% (15/49) in the RT group, 22.2% (4/18) in the CRT group, and 15.0% (3/20) in the RIT ± CT group, with the RIT ± CT group showing the lowest rate. No statistically significant differences in locoregional recurrence, distant metastasis, or mixed failure patterns were observed across the three groups (all P > 0.05), likely due to the limited sample size.

### 3.5 Propensity score matching analysis (RT vs. RIT ± CT)

**Figure 3:**
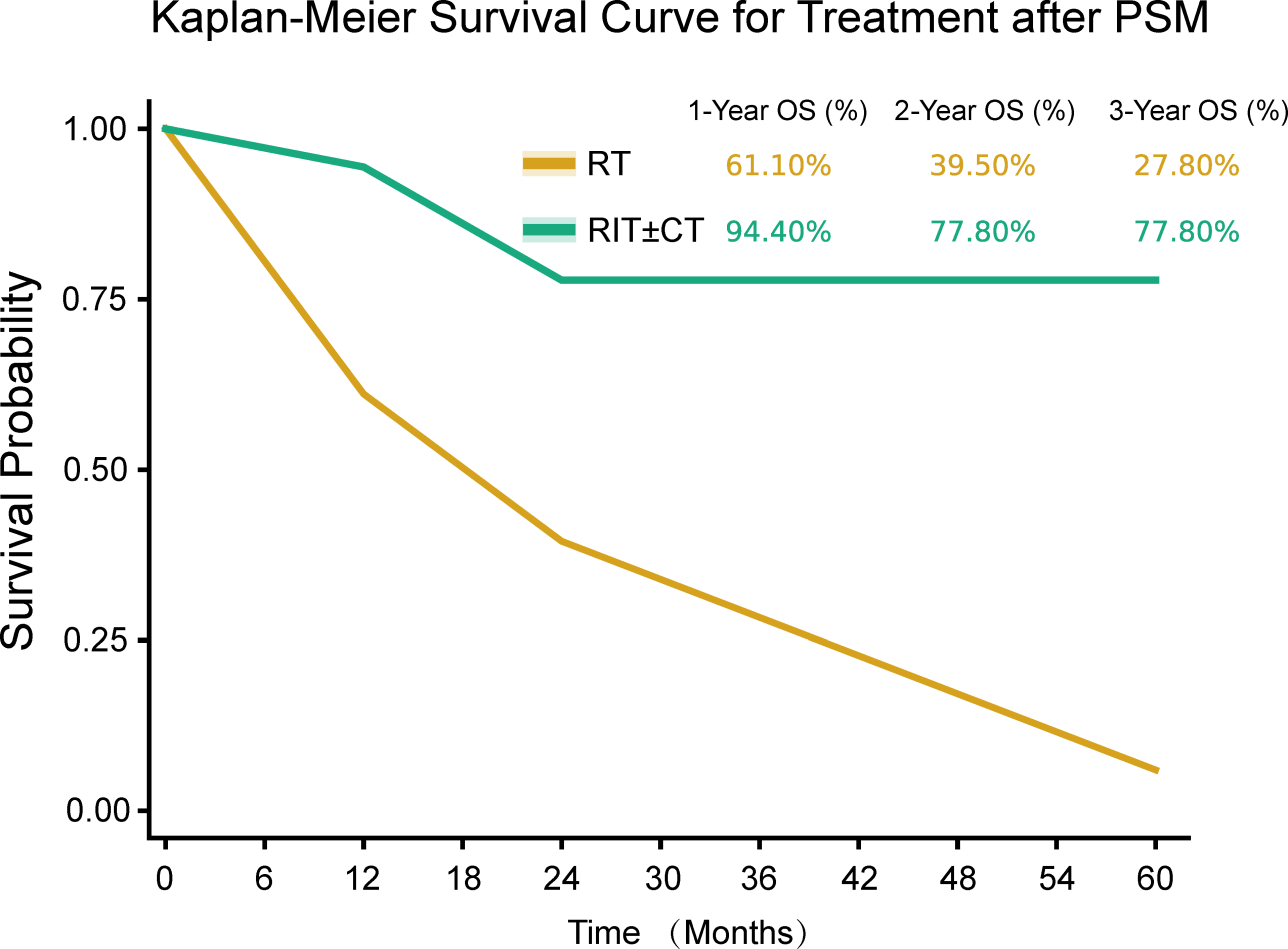
Kaplan–Meier survival curves by treatment group after propensity score matching. Overall survival after propensity score matching (RT in yellow, RIT±CT in green). Log-rank P = 0.009.

After 1:1 matching, 36 patients (18 per group) demonstrated well-balanced baseline characteristics, with all standardized mean differences <0.1 (**Table 6**). RIT ± CT maintained a significant OS benefit versus RT (unadjusted HR = 0.39, 95% CI: 0.18–0.84, P = 0.012) and PFS advantage (unadjusted HR = 0.42, 95% CI: 0.19–0.91, P = 0.028). RIT ± CT 1-, 2-, and 3-year OS rates were 94.4%, 77.8%, and 77.8%, respectively, significantly higher than RT (61.1%, 38.9%, and 27.8%; log-rank P = 0.009). The consistency of results between multivariable analysis and PSM, despite the small sample, strengthens the internal validity of the finding but does not overcome the limitation of small absolute patient numbers. Multivariable Cox regression based on the matched cohort (n=36) confirmed RIT ± CT’s independent OS association (adjusted HR = 0.42, 95% CI: 0.19–0.91, P = 0.028). Higher radiation dose remained protective (adjusted HR = 0.963, 95% CI: 0.941–0.986, P = 0.002), and tumor length >5 cm remained a risk factor (adjusted HR = 1.276, 95% CI: 1.065–1.529, P = 0.008). Results of the PSM analysis are shown in **Tables 6**, **7**, **Figure 3**.

**Table 6:** Comparison of baseline characteristics before and after propensity score matching (RT vs. RIT ± CT).

Variable	Before PSM		SMD	After PSM		SMD
	RT	RIT ± CT		RT	RIT ± CT	
Age (years)	77.2	75.8	0.32	75.9	75.8	0.08
Male, %	63.3	60.0	0.12	61.1	61.1	0.00
BMI (kg/m²)	21.5	22.3	0.15	22.0	22.2	0.04
Tumor length >5 cm, %	44.9	55.0	0.14	50.0	50.0	0.00
Clinical stages III-IV, %	57.1	60.0	0.18	55.6	55.6	0.00
Total CIRS-G score	5.2	5.8	0.25	5.7	5.8	0.03
Medium CIRS-G burden, %	38.8	40.0	0.02	38.9	38.9	0.00

CRT group was not included in PSM.

**Table 7:** Multivariable Cox regression analysis in the matched cohort (RT vs. RIT ± CT, n=36).

Variable	Hazard ratio (HR)	95% confidence interval (95% CI)	P-value
**RIT ± CT group (Ref: RT group)**	**0.42**	**0.19–0.91**	**0.028**
Age (per 1-year increase)	1.038	0.974–1.106	0.247
**Tumor length (>5 vs. ≤5 cm)**	**1.276**	**1.065–1.529**	**0.008**
**Radiation dose (per 1-Gy increase)**	**0.963**	**0.941–0.986**	**0.002**

This model was based on the matched cohort of 36 patients (RT, n=18; RIT ± CT, n=18) and adjusted for age, tumor length, and radiation dose. The CRT group was not included in PSM and thus not analyzed here. Bold values indicate statistical significance (P < 0.05).

### 3.6 CIRS-G stratification subgroup analysis

**Figure 4:**
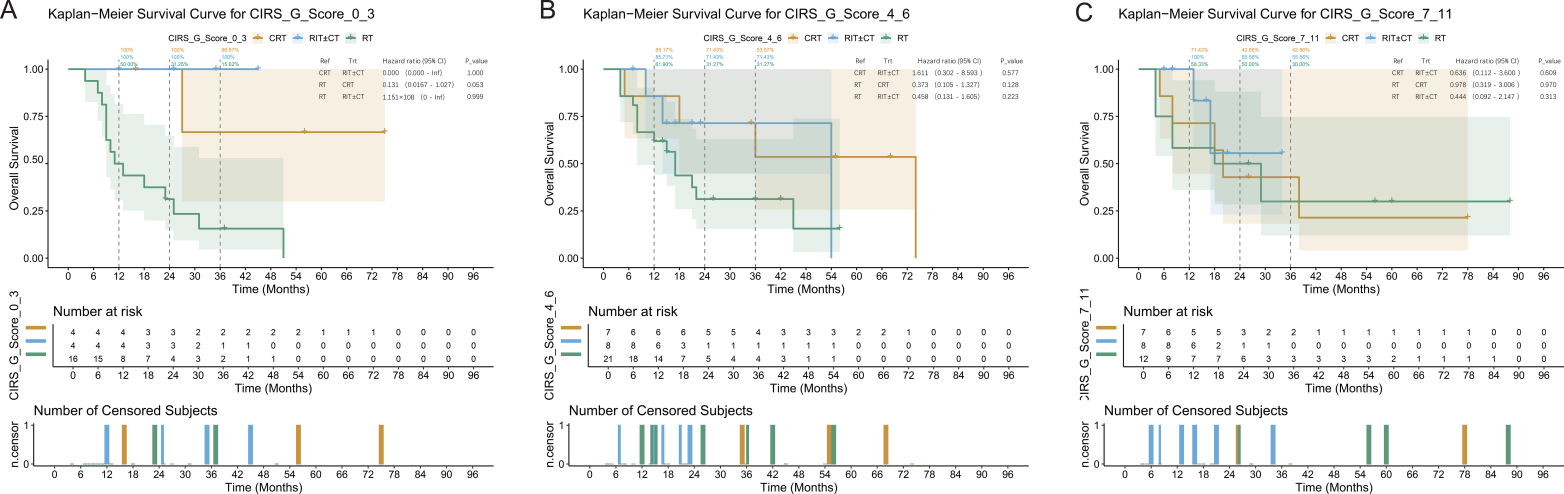
Subgroup survival analysis based on CIRS-G stratification in 87 patients. Subgroup survival by CIRS-G burden: low (left), medium (middle), and high (right). Log-rank P-values are shown.

Although the total CIRS-G score was not an independent prognostic factor in the overall cohort, heterogeneous treatment responses were observed across comorbidity burden subgroups. Low Burden (CIRS-G 0–3, n=24): The small sample size in this subgroup limited meaningful statistical inference. Medium Burden (CIRS-G 4–6, n=36): The RIT ± CT group (n=8) exhibited the most favorable OS trend, with a 3-year rate of 71.43%, compared with 53.57% in the CRT group and 31.27% in the RT group. In this subgroup, the HR of 0.36 (95% CI: 0.14–0.92) should be viewed as a signal for further investigation rather than a definitive effect estimate. PSM-based subgroup analysis further confirmed a significant survival benefit with RIT ± CT in these patients (HR = 0.36, 95% CI: 0.14–0.92, P = 0.033). High Burden (CIRS-G ≥7, n=27): No statistically significant differences in survival were observed among the treatment groups (log-rank P = 0.616), and RIT ± CT did not yield a significant OS benefit (HR = 0.44, 95% CI: 0.09–2.15, P = 0.313). Corresponding subgroup survival curves are provided in **Figure 4**.

### 3.7 Safety analysis

TRAEs demonstrated a clear treatment–intensity correlation. Most were grades 1–2 and clinically manageable. Grade ≥3 TRAEs occurred most frequently in the RIT ± CT group (55.0%, 11/20), primarily hematologic toxicity (55.0%), followed by radiation pneumonitis (20.0%), and radiation esophagitis (10.0%). The incidence of grade ≥3 TRAEs was 38.9% (7/18) in the CRT group and 20.4% (10/49) in the RT group. Two RIT ± CT patients (10.0%) developed immune-related pneumonia (grade 2), which resolved after temporary immunotherapy interruption and corticosteroids. All grade ≥3 adverse events were controlled with supportive measures. No treatment-related deaths occurred. A comprehensive summary of treatment-related toxicities is provided in **Table 8**.

**Table 8:** Treatment-related acute toxicities in 87 elderly patients with ESCC.

Item	RT group (n=49)	CRT group (n=18)	RIT ± CT group (n=20)	Total (n=87)	χ² value	P-value
Grade ≥3 hematologic toxicity	1 (2.05%)	3 (16.7%)	11 (55%)	15 (17.24%)	28.67	<0.001
Grade ≥3 radiation esophagitis	3 (6.12%)	1 (5.5%)	2 (10%)	6 (6.89%)	0.45	0.799
Grade ≥3 radiation pneumonitis	6 (12.2%)	3 (16.7%)	4 (20%)	13 (14.94%)	0.78	0.677
Immune-related pneumonia*	—	—	2 (10%)	2 (2.3%)	—	—

*Immune-related pneumonia occurred only in the RIT ± CT group (2/20, 10.0%). Between-group statistical comparison was not performed because the event was absent in the RT and CRT groups.

## 4 Discussion

This real-world study demonstrates that radiotherapy combined with immunotherapy-based regimens (mostly with chemotherapy) is associated with significantly improved survival in elderly ESCC patients with a manageable safety profile. Notably, our CIRS-G-based stratification revealed a non-linear relationship between comorbidity burden and therapeutic benefit, with patients of moderate burden (scores 4–6) exhibiting the most substantial advantage.

Our efficacy results align with growing evidence from several studies reporting survival benefits with immunotherapy combined with chemoradiotherapy in esophageal cancer (14–17). However, it is important to note that a recent multicenter study reported that adding anti-PD-1 antibodies to definitive chemoradiotherapy did not result in significant OS or PFS improvement in elderly ESCC patients overall, although exploratory findings suggested potential benefits in selected patients with favorable baseline profiles (18). Our findings extend these observations by demonstrating that CIRS-G-based comorbidity stratification may help identify those “selected patients” who derive benefit, particularly those with medium comorbidity burden.

The 65.9% reduction in mortality risk with RIT ± CT versus radiotherapy alone remained significant after rigorous propensity score matching, confirming the robustness of our findings. Furthermore, the significant positive correlation between radiation dose and survival benefit reinforces the established dose–response relationship in radiation oncology (13), and mechanistic studies have further elucidated the complex interplay between radiation dose, tumor microenvironment, and resistance pathways (19).

Interestingly, conventional prognostic factors including age, AJCC stage, and tumor location failed to demonstrate independent prognostic value in our multivariate model. This suggests that their influence may be superseded by stronger predictors such as treatment modality and comorbidity burden in this specific elderly population. The differential benefit across comorbidity strata may also be explained by the interplay between immunosenescence and chronic inflammation (3, 20). Older adults with higher comorbidity burdens often exhibit a more profoundly immunosuppressive tumor microenvironment (21). Emerging evidence also suggests that intratumoral microbiota may influence cancer initiation and therapeutic efficacy (20), although its role in elderly ESCC requires further investigation.

Regarding the patterns of failure, although no statistically significant differences were observed among the groups (likely due to the limited sample size), a notable trend emerged. The RIT ± CT group exhibited the lowest rate of distant metastasis (15.0%) compared with the RT (30.6%) and CRT (22.2%) groups. This trend suggests that the addition of immunotherapy to radiotherapy may provide improved systemic disease control, potentially through an “abscopal effect” synergy (8), where radiotherapy releases tumor antigens and immunotherapy enhances systemic immune surveillance, thereby mitigating the risk of distant dissemination.

From a safety perspective, the manageable toxicity profile observed in our RIT ± CT group aligns with the established principles of toxicity management in trimodal therapy, as demonstrated in large trials like JCOG1109 (22). Although the incidence of grade ≥3 TRAEs was higher in the RIT ± CT group (55.0%), these events were primarily hematologic toxicities that were effectively managed with standard supportive measures, and no treatment-related deaths occurred. Moreover, the use of immune checkpoint inhibitors in older adults should be guided by geriatric assessment, as recommended by recent literature (23).

Unlike current research focusing on concurrent combinations or maintenance approaches (22, 24–27), our sequential strategy—featuring initial tumor debulking and microenvironment modulation prior to radiotherapy—may optimize the therapeutic window for radiation. This approach may also potentially enhance treatment tolerance through temporal separation of therapeutic modalities.

The CIRS-G-guided treatment stratification proposed here carries immediate clinical relevance. For patients with CIRS-G scores ≤6, active combination therapy should be strongly considered. For those with scores ≥7, careful weighing of the efficacy–toxicity balance is warranted, with prioritization of better-tolerated regimens. This comorbidity-based triage system represents a practical application of precision medicine principles in geriatric oncology, moving beyond chronological age to incorporate biological vulnerability into therapeutic decision-making.

### 4.1 Important caveats must be emphasized

First, 85% of patients in the RIT ± CT group received concurrent chemotherapy, making it impossible to isolate the independent contribution of immunotherapy. The observed survival benefit likely results from the intensified multimodal regimen (radiotherapy + immunotherapy + chemotherapy) rather than immunotherapy alone. Therefore, our title and conclusions have been carefully worded to reflect this “immunotherapy-based combination” rather than pure immunotherapy add-on.

## 5 Limitations

Several limitations should be considered when interpreting our findings. First, the single-center retrospective design introduces potential selection bias despite statistical adjustments using propensity score matching. Second, the modest sample size, particularly in subgroup analyses, may limit statistical power for detecting more subtle treatment effects. Third, the absence of patient-reported outcomes and quality-of-life assessments restricts our understanding of the full impact of treatment intensification on elderly patients’ lived experiences. Fourth, the RT group had a higher proportion of patients aged ≥80 years compared with the CRT and RIT ± CT groups (46.9% vs. 11.1% and 20.0%, respectively), which may have contributed to its poorer survival outcomes. Although age was adjusted for in multivariate and PSM analyses and did not retain independent prognostic significance, residual confounding from this baseline imbalance cannot be entirely excluded.

Most importantly, the inability to attribute the observed benefit to immunotherapy alone—due to concurrent chemotherapy in 85% of RIT ± CT patients—represents a fundamental limitation. Future studies with cleaner exposure definitions (e.g., RT+IO without concurrent chemotherapy) are needed to validate the independent contribution of immunotherapy.

## 6 Conclusion

This exploratory real-world study suggests that radiotherapy combined with immunotherapy-based regimens (with or without chemotherapy) may be an effective strategy with a manageable safety profile for elderly ESCC patients. The CIRS-G score shows promise as a tool for identifying optimal candidates, specifically those with medium comorbidity burden (scores 4–6). For high comorbidity burden patients (CIRS-G ≥7), careful risk–benefit assessment is recommended. Given the modest sample size, particularly in the immunotherapy-containing group (n=20), these findings should be considered hypothesis-generating. The independent effect of immunotherapy alone cannot be determined. Prospective, larger-scale studies are urgently needed to validate these observations.

## Data Availability

The original contributions presented in the study are included in the article/supplementary material. Further inquiries can be directed to the corresponding author.
